# An Optimized Ensemble Deep Learning Model for Predicting Plant miRNA–IncRNA Based on Artificial Gorilla Troops Algorithm

**DOI:** 10.3390/s23042219

**Published:** 2023-02-16

**Authors:** Walid Hamdy, Amr Ismail, Wael A. Awad, Ali H. Ibrahim, Aboul Ella Hassanien

**Affiliations:** 1 Faculty of Science, Port Said University, Port Said 42511, Egypt; 2 Faculty of Computers and Artificial Intelligence, Damietta University, El-Gadeeda 34519, Egypt; 3 Faculty of Computers and Artificial Intelligence, Cairo University, Giza 12613, Egypt

**Keywords:** CNN model, deep learning, ensemble learning, lncRNA, IndRNN, microRNA, plant

## Abstract

MicroRNAs (miRNA) are small, non-coding regulatory molecules whose effective alteration might result in abnormal gene manifestation in the downstream pathway of their target. miRNA gene variants can impact miRNA transcription, maturation, or target selectivity, impairing their usefulness in plant growth and stress responses. Simple Sequence Repeat (SSR) based on miRNA is a newly introduced functional marker that has recently been used in plant breeding. MicroRNA and long non-coding RNA (lncRNA) are two examples of non-coding RNA (ncRNA) that play a vital role in controlling the biological processes of animals and plants. According to recent studies, the major objective for decoding their functional activities is predicting the relationship between lncRNA and miRNA. Traditional feature-based classification systems’ prediction accuracy and reliability are frequently harmed because of the small data size, human factors’ limits, and huge quantity of noise. This paper proposes an optimized deep learning model built with Independently Recurrent Neural Networks (IndRNNs) and Convolutional Neural Networks (CNNs) to predict the interaction in plants between lncRNA and miRNA. The deep learning ensemble model automatically investigates the function characteristics of genetic sequences. The proposed model’s main advantage is the enhanced accuracy in plant miRNA–IncRNA prediction due to optimal hyperparameter tuning, which is performed by the artificial Gorilla Troops Algorithm and the proposed intelligent preying algorithm. IndRNN is adapted to derive the representation of learned sequence dependencies and sequence features by overcoming the inaccuracies of natural factors in traditional feature architecture. Working with large-scale data, the suggested model outperforms the current deep learning model and shallow machine learning, notably for extended sequences, according to the findings of the experiments, where we obtained an accuracy of 97.7% in the proposed method.

## 1. Introduction

Eukaryotic genomes produce non-coding RNAs (ncRNAs) with broad transcription features, and researchers discovered that only around 1–2% of transcripts are engaged in coding the protein [1]. Non-coding RNAs (ncRNAs) are most transcripts that are not implicated in protein coding [2]. In the past decade, the number and ncRNA function of species have become important research topics in biology. ncRNA is categorized as short non-coding RNA (sncRNA) or long non-coding RNA (lncRNA) depending on if the length of the transcript is greater than 200 nt [3]. The most common kinds of this sncRNA are lncRNA and miRNA [4]. The increasing knowledge of function ncRNA technicality has the attention of a growing number of researchers.

Detection and function investigation of sncRNA has become a popular topic. Additionally, the relationship between lncRNA and miRNA, according to researchers, is significant in the control of gene expression and is linked to species development, material metabolism, embryonic development, and the incidence of numerous disorders [5]. As a result, it is critical to understand how RNA molecules interact and how they operate. A thorough investigation of the interplay between lncRNA and miRNA will transform our present comprehension of cell structures and organization and provide significant scientific and medicinal benefits.

In plants, there are two kinds of interactions between lncRNA and miRNA. (1) In addition to performing a direct role as a miRNA precursor, lncRNA can be spliced into shorter miRNA. For example, MiR869a and miR160c may be spliced into lncRNAs npc521 and npc83 [6]. (2) lncRNA can be cleaved by miRNA as a target [7]. By functioning as a target for miRNA, lncRNA controls the phosphate balance in plants in vivo by reducing miRNA’s inhibitory impact on genes. The “sponge effect” [8] describes how lncRNA might operate as a miRNA decoy, competing with mRNA for miRNA binding and thereby regulating the expression of miRNA target genes. In tomato yellow mosaic virus (TYMV), two lncRNAs, slylnc1077 and slylnc0195, were discovered to behave as decoys for miRNAs [9]. Slylnc0195 expression is up-regulated in tomatoes that are infected with TYMV; however, miR166a expression is downregulated.

According to studies, the relationship between lncRNA and miRNA regulates plant disease impedance, cell differentiation, vernalization, fruiting and flowering, drought resistance, cold tolerance, and other biotic and abiotic stress resistance mechanisms [10]. There are fewer investigations on the relationship between lncRNA and miRNA in plants than in human and animal studies. Furthermore, only a few lncRNA and miRNA mechanisms of action have been verified, resulting in a lack of experimental data on lncRNA and miRNA in the area of plants, making bioinformatics criteria for in-depth analysis of the interactions between lncRNA and miRNA difficult to satisfy. Thus, obtaining a vast amount of data about the interactions of lncRNA and miRNA in relation to plant growth and development is critical for in-depth research of the functional mechanism of lncRNA and miRNA interaction in plants.

To anticipate the interactions between lncRNA and miRNA, at the moment, shallow machine learning is used in many articles to structure systems for prediction through features selected in the prediction of the interactions between lncRNA and miRNA. However, there are numerous issues such as a lack of training data, human factors, and large noise, which together result in reduced predictability of the results. In recent years, the development of computational approaches for finding connections in different biological datasets has received much attention [11]. This approach employs a two-layer Convolutional Neural Network (CNN) [12] to understand sequence characteristics and discover functional domains in nucleotide sequences. After that, a two-layer IndRNN is used to understand long-term functional domain dependences to categorize data [13].

The relationship between miRNA and lncRNA has been shown in studies to have a key regulatory function in plant disease resistance, vernalization, cell differentiation, flowering and fruiting, cold tolerance, drought resistance, and other biotic and abiotic stressors. In comparison to people and animals, there have been few studies on the interplay between miRNA and lncRNA in plants. Furthermore, only a few miRNA and lncRNA modes of action have been verified, resulting in a lack of experimental data on miRNA and lncRNA in plants, making it difficult to meet bioinformatics requirements for in-depth investigation of the interaction between miRNA and lncRNA. As a result, a vast amount of data on the interaction between miRNA and lncRNA related to plant growth and development is critical for in-depth research of the functional mechanism of the interaction between miRNA and lncRNA in plants. Predicting the link between lncRNA and miRNA is the primary goal of deciphering their functional activities. The prediction accuracy and reliability of traditional feature-based classification systems are commonly injured due to small data size, human factor limitations, and a large amount of noise. According to recent studies, it was found that when any error, even a slight one, occurs in the predicted relationship between lncRNA and miRNA, this leads to significant problems in the plant classification. This inspired our research, seeking to improve the performance of the prediction relationship between lncRNA and miRNA, which play vital roles in controlling the biological processes of animals and plants. The study’s contributions are summarized as follows:

- We propose CNN-RNN, a deep learning model to predict the interaction in plants between lncRNA and miRNA.
- The GTO algorithm is used to learn the parameters of the CNN-RNN. As a result of the GTO’s convergence performance, the prediction success of CNN-RNN with GTO has been increased.
- We lay the groundwork for further investigation into the interaction mechanism of miRNA and lncRNA in plants.

## 2. Related Work

Deep learning has recently been popular in a variety of miRNA datasets, as it outperforms traditional methods in miRNA prediction. For prediction, researchers used either pre-trained models or ordinary deep learning [14].

LncRNAs and miRNAs have been demonstrated to control each other. Zhang et al. [15] suggested “M6A-HPCS”, which enhanced the accuracy of m6A prediction by introducing a new methodology, the nucleotide physical–chemical property selection (HPCS) algorithm. Zhou et al. [16] developed SRAMP, which predicted m6A locations in humans and other animals such as mice using sequence features and the random forest (RF) approach. This approach generated three RF classifiers using KNN encoding, binary encoding, and spectrum encoding. Finally, a weighted sum was used to combine the three categorization models into a single model. However, the accuracy of m6A location predicted on the benchmark yeast datasets was not as good as that of the benchmark mammalian datasets using this strategy. Chen et al. [17] presented the “MethyRNA” technique in 2017, which used the SVM algorithm to classify RNA sequences and sequence-derived feature coding. Qiang et al. [18] suggested the “M6AMRFS” framework, which coupled the Sequential Forward Search (SFS) algorithm and the F1-score to enhance the ability of feature representation and used the XGBoost algorithm to develop a new method of feature representation that uses two features described for RNA code sequences. Authors in [19] suggested “M6Apred-EL” using ensemble learning in the same year. Three SVM classifiers were trained using this technique, which looked at ring function hydrogen chemical properties, physical–chemical information, physical–chemical characteristics, and position-specific data. For the first time, Ref. [20] presented the “DeepM6ASeq” approach, which combined CNN and BLSTM networks with one-hot encoding. Zou et al. [21], in 2019, developed the deep learning model “Gene2vec”. This technique considered the influence of RNA sequence prediction window length on experimental accuracy. It addressed four potential sequence encoding methods and, for the first time, used the word2vec model for RNA encoding sequences. Ref. [22] introduced the “WHISTLE” model in this year, which merged RNA sequence characteristics and genomic features and categorized the sequences using the SVM method; this model increased the m6A site performance of prediction (average accuracy: 94.8% and 88.0% under mature mRNA models and the whole transcript, respectively). Liu et al. [23] created a systematic approach that can concurrently identify m6A, m5C, m1A, and Ato-I adjustments in several species. The majority of the approaches listed above are based on standard methods of machine learning, which lack deep learning exploration and primarily concentrate on feature extraction, resulting in poor model generalizability and prediction accuracy.

A novel meta-heuristic Artificial Gorilla Troops Optimizer (GTO) technique was suggested recently [24]. GTO displays gorilla social behavior. It effectively solves mathematical problems, producing optimal solutions in a fraction of the time required by other leading techniques. GTO has recently been used to tackle optimization problems in a variety of fields. GTO was employed by Ahmed Ginidi et al. [25] for photovoltaic model parameter extraction, while Abdel-Basset et al. [26] presented memory-based improved GTO (MIGTO) for the same problem. To handle feature selection in biological data, Piri et al. [27] introduced discrete artificial GTO (DAGTO). Liang et al. [28] updated GTO with opposition-based learning and parallel methods, offering OPGTO to decrease errors in the wireless sensor network’s node location. For the problem of global optimization, Xiao et al. [29] presented enhanced GTO based on two strategies, namely lens opposition-based learning and adaptive *β*-hill climbing.

Essam et al. [30] discusses how the Gorilla Troops Algorithm is used to minimize the energy consumption of large-scale wireless sensor networks. As for the energy usage scores, optimum sink node placements and localization faults are addressed to carefully examine the efficacy of the considered MO approaches and determine the optimal positions and the smallest number of sink nodes that will satisfy the entire network. Authors show that the MOGTO model allocated the smallest sink nodes across all network sizes, demonstrating its efficacy in reducing energy consumption averages and increasing the network’s lifetime. Wu, Tingyao et al. [31] proposed a new Modified Gorilla Troops Optimizer (MGTO). There are three aspects to the improvement strategies: Quasi-Reflection-Based Learning (QRBL), Beetle Antennae Search Based on Quadratic Interpolation (QIBAS), and Teaching–Learning-Based Optimization (TLBO). They compared the MGTO with traditional GTO, SSA, GWO, ROLGWO, PSO, WOA, AOA, HSCAHS, and DSA algorithms to prove that MGTO has efficiency and promising potential in real-world optimization challenges. El-Dabah et al. [32] investigated how well a power system stabilizer (PSS) unit may be modified by using the GTO algorithm. They used the Integral Time Square Error (ITSE) as a fitness function that should ideally be minimized. They also used four alternative controllers to study a single machine scheme model as a model for the infinite bus. They determined that the GTO algorithm has faster convergence over the other compared optimization techniques. Bhadoria et al. [33] proposed a Chaotic Gorilla Troops Optimizer (CGTO) to present a novel solution to the power generation scheduling problem. The gorilla update technique first collects a binary string of generators in order to find the global best solution (s), and then performs a chaotic operation. Finally, they reported that the CGTO has a strong performance compared with other various techniques for solving the power generation scheduling problem.

## 3. CNN’s Model Setting and Phases

Initially, shallow machine learning approaches depending on feature engineering were used to classify and predict issues. However, due to their numerous disadvantages, researchers have begun to focus on deep learning methods [34]. Deep learning has recently become popularly employed for sequence classification [35], biological information [36], image processing [37], computer vision [38], natural language processing [39], and other sectors, with positive outcomes.

### 3.1. Structure of IndRNN and CNN

**Figure 1:**
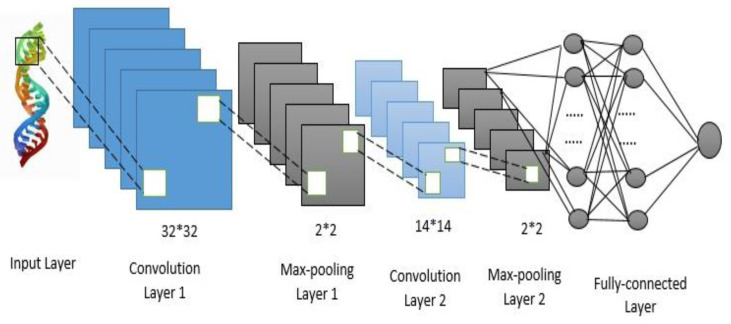
A Convolutional Neural Network (CNN) frame structure.

CNNs and recurrent neural networks (RNN) are the most common deep learning models [40]. Their variations make up the majority of existing deep-learning models. CNN-RNN is the combination of IndRNN and CNN. The model employs a two-layer CNN to extract significant features from the best filter. The CNN convolution layers extract data feature information on multiple levels [41] and process the feature during the pooling layers to obtain the best classification features. The collected feature information is then passed to the layers of IndRNN, which uses it to learn more about the feature dependencies. A dropout layer is used in the model to avoid overfitting. Simultaneously, the ReLU function was chosen as the function activation since it outperforms the sigmoid function in terms of promoting sparse and successfully decreasing the gradient likelihood value [42]. Figure 1 depicts the specific construction of the ensemble CNN.

**Figure 2:**
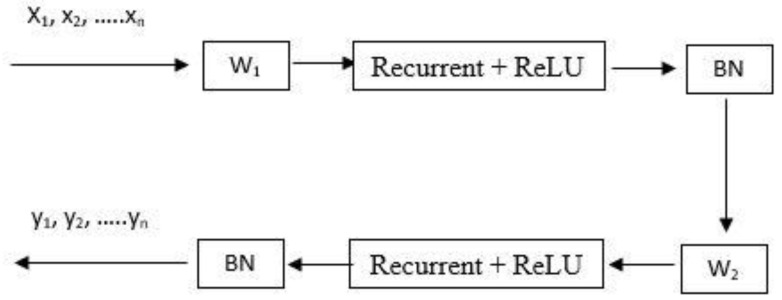
Independently Recurrent Neural Network (IndRNN) frame structure.

Long-term reliance between sequences can be learned by IndRNN. The model employs a two-layer IndRNN structure to learn sequence dependencies better. IndRNN is a simple structure that may be readily expanded to many network designs, unlike classic RNN. Because neurons in the same layer are autonomous, each neuron’s behavior can be studied without considering the impact of another neuron. It can tackle the gradient explosion and gradient disappearance problems in standard RNNs by deepening the network level without sacrificing the ability to deliver the training loop or rely on gateway parameters [43] while still preserving long-term memory. As a result, the gradients can be successfully transmitted in various time steps. Many IndRNNs can be stacked together to build a bigger network, allowing the network to be more in-depth and persistent, investigating information across channels and understanding data dependency. Equation (1) can describe the update status as follows:

(1) $$h_{t}=\;\sigma \left(W_{x_{i}}+U⊙h_{t-1}+b\right)$$

where *x_t_* and *h_t_* denote the inputs and hidden states, respectively, at timestep *t*. *U* and *W* are recurring inputs and the weights of the currents, respectively, while b is the neuron’s bias. *W*_1_, *W*_2_, and ReLU and Recurrent represent the loop processing and input weights for each step, with ReLU as the activation function, and **BN** signifies standardized batch processing. As shown in Figure 2, further deepening of the IndRNN model can be achieved by layering this structure.

### 3.2. Ensemble CNN (CNN and IndRNN)

We propose a new model, CNN-RNN, that depends on a deep learning ensemble basis of traditional IndRNN and CNN. The model is divided into two halves. The classic CNN is a feed-forward neural network that uses convolution to extract features and then pool layers to understand local input data characteristics. Another component is IndRNN, which is an RNN expansion. Internal feedback connection, internal memory, and feed-forward adjustment between processing components are properties of RNN. As a result, it positively impacts the processing of sequence information.

**Figure 3:**
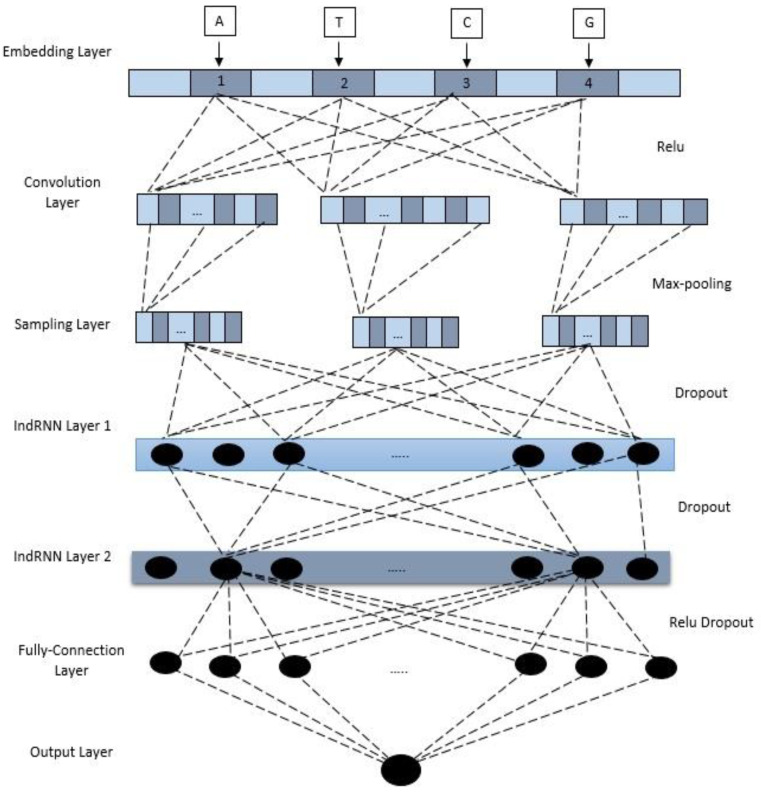
The general architecture of the proposed model.

On the other hand, CNN ignores the connection between non-continuous sequences and only looks at the connection between continuous sequences when dealing with sequence data. Although RNN is well suited to processing sequence data, it is challenging to address long-term information dependency. There are also issues with gradient disappearance and gradient explosion. CNN-RNN combines the strengths of CNN and IndRNN. This allows for the comprehensive extraction of feature information and the consideration of long-term sequence dependence. Figure 3 depicts the general architecture of the ensemble model.

## 4. Artificial Gorilla Troops Optimizer (GTO)

The Artificial Gorilla Troops Optimizer (GTO) was recently described as a metaheuristic optimization method based on gorilla behavior [24]. In solving numerous engineering issues, the GTO optimizer demonstrated remarkable accuracy and efficiency [25]. Furthermore, by raising the number of search capabilities, the GTO method has an extraordinary capacity to produce desirable outcomes and acceptable performance for various system dimensions. It also outperforms other optimizers in all similar dimensions because other optimizers’ efficiency degrades as the number of dimensions increases. Another benefit of the GTO is that it excels at balancing exploration and exploitation skills in the face of large-scale problems [24].

During this phase, each gorilla is set up as a competitor for the best solution in each iteration, with the best solution being known as the silverback gorilla. Three different mechanisms are summarized in Equation (2).

(2) $$GX\left(t-1\right)=\{\begin{array}{ll} \left(U_{r}-L_{r}\right)r_{1}+L_{r} & if\;rand<0\\ \left(r_{2}-D\right)X_{r}\left(t\right)+S\times V & if\;rand\;\geq \;0.5\\ X\left(i\right)-S\left(S\left(X\left(t\right)-GX_{r}\left(t\right)\right)+r_{3}\left(X\left(t\right)-GX_{r}\left(t\right)\right)\right) & if\;rand<0.5 \end{array}$$

where *O* is the parameter for migration to an unknown site. *GX*(*t* − 1) represents the candidate of the gorilla vector position in the next iteration, rand is a random variable, *X*(*t*) represents the gorilla position of the current vector, and *X*(*i*) represents the candidate gorilla member number [44]. Furthermore, *Lr* and *Ur* denote the lower and upper limits of the problem variables, respectively. *GX_r_*(*t*) denotes the position of one of the randomly picked gorilla candidates, and *X_r_*(*t*) represents the position of this random gorilla. Furthermore, the random variables *r*_1_, *r*_2_, and *r*_3_ fall in the range [0, 1]. The following equations are used to compute the parameters *D*, *V*, and *S*.

(3) D=N×(1−t∕MaxIt)

(4) S=D∗R

(5)  V=T/X(i)

where *MaxIt* is the maximum number of iterations, *R* is the random number in the range [−1, 1], and *T* is the random value in the range [*−D*, *D*]. The male gorillas in the group are accustomed to following the silverback to find food. A “silverback” is the optimal solution found during this step. This behavior can be expressed quantitatively as follows:

(6) $$G_{x}\left(t+1\right)=S*{\left(|\;1\backslash N\sum_{J-1}^{N}G_{j}\left(t\right)|\right)}^{1\backslash 2^{S}}$$

while the choice of adult females can be mathematically stated as follows:

(7) $$G_{x}\left(t+1\right)=Y_{\mathrm{silverback}}-\left(Y_{\mathrm{silverback}}-Y\left(t\right)\right)*\left(2*rand-1\right)*A$$

where $Y_{\mathrm{silverback}}$ is the best candidate solution’s position (silverback), and *A* is a constant parameter.

Finally, at the end of the exploitation phase, the fitness function solution is updated with the best solution. The following are the main steps of the GTO Algorithm 1:

1. Create a population with random positions.
2. Set the parameters D, S, L, U, and MaxIt.
3. Determine the gorilla’s position using Equation (2).
4. Evaluate each gorilla’s fitness function.
5. Set the best solution as the silverback’s location.
6. Update the gorilla position based on D and W values using Equation (7).
7. Display the best gorilla posture and keep the fitness function updated until the maximum number of iterations is reached.

For evaluating the fitness function score for each solution, as previously stated, each solution is composed of random floating point values between 0 and 1. A fitness/objective function is required to evaluate the individual solution in feature selection (FS). The primary goal of feature selection is to enhance forecast accuracy while lowering the number of features. More specifically, in this variation of the proposed work, the objective function (*OF*), which combines both criteria, is described as

(8) $$OF\left(O\right)=\alpha *\;classification_{error}+\left(1-\alpha \right)*\frac{LS}{L}\;$$

where *classification_error_* is the learning algorithm’s error rate, *LS* is the length of the feature substring, *L* is the original dimension, and α (here, 0.99) is a control parameter for the effect of classification performance and feature size.
**Algorithm 1.** contains the detailed algorithm for the proposed GTOAlgorithm for the proposed GTO1for all Gorilla Oi do2Alter the gorilla location by Equation (1)3Use the sigmoid function to turn the gorilla location into a probability value4Using Equation (6), for compute the candidate gorilla position in the discrete domain5end for6for I = 1to N do7Compute OF of each candidate gorilla (Gi)8If Gi is fitter than Oi, replace it, 9end for10Set the best location as the Silverback11for all Gorilla Oi do12if D ≥ W then13Using Equation (7) for changing the Gorilla’s location14Else15Using Equation (6) for changing the Gorilla’s location16end if17end for18  for I = 1to N do19  Compute OF of each candidate gorilla (Gi)20Replace Oi if Gi is more appropriate, where G is the candidate Gorilla location21end for22Set best location as the Silverback23end for

## 5. Experiment

First, data preparation was performed before training the model. A, T, C, and G were represented by the numbers 1, 2, 3, and 4, respectively. The data were subsequently transformed into a matrix by the embedding layer, then delivered to CNN. The convolution operations were used to extract the feature information via the convolution layer, while the maximum pooling operation was used to filter out the significant local feature information. After the ReLU function was activated, the vector matrix was turned into a features map, used as one of IndRNN’s input layers. IndRNN was used to understand the relationship between features thoroughly. Lastly, the predicted results were obtained by translating the vector features for the output of IndRNN to a concrete numbering using the dense layers and mapping the number to [0, 1] using the sigmoid function. The BP method [45] was used for calculating the loss layer by layer to update the parameter based on the difference between the real and forecasted values. To avoid overfitting, we used a dropout layer with a value of 0.5. The model’s learning rate was set to 0.01, the batch size was 128, and the model was optimized using the Artificial Gorilla Troops Optimizer (GTO), as shown in Table 1.

## 6. Evaluation Phase

In this stage, five evaluation indicators are often used in classification issues and were used here to assess the predictive strength of the proposed approach: specificity, accuracy, precision, F1-score, and recall. Specificity is defined as the conditional probability of actual negatives having a secondary class, equating to the likelihood of a negative mark being true, and it’s calculated using Equation (9). Accuracy is the percentage of validation predictions made for all forecasts, usually expressed as percentages and determined using Equation (10). Precision is a metric that assesses a model’s ability to correctly forecast the value for specific categories and is calculated using Equation (11). Recall measures the proportion of correctly identified positive patterns, determined using Equation (12). The F1-score is the average weight of recall and precision calculated with Equation (13).

(9) $$\mathrm{Specificity}\;=\frac{TN}{TN+FP}$$

(10) $$\mathrm{Accuracy}\;=\frac{TP+TN}{TP+TN+FP+FN}$$

(11) $$\mathrm{Precision}\;=\frac{TP}{TP+FP}$$

(12) $$\mathrm{Recall}\;=\frac{TP}{TP+FN}$$

(13) $$F1-\mathrm{score}\;=2*\;\frac{\mathrm{Precision}*\;\mathrm{Recall}}{\mathrm{Precision}\;+\;\mathrm{Recall}}$$

where *TP* denotes the positive number of positively predicted classes, *TN* denotes the negative number of negatively predicted classes, *FN* denotes the positive number of negatively predicted classes, and *FP* denotes the negative number of classes that are positively predicted.

## 7. Results and Discussion

### 7.1. Dataset Description

**Figure 4:**
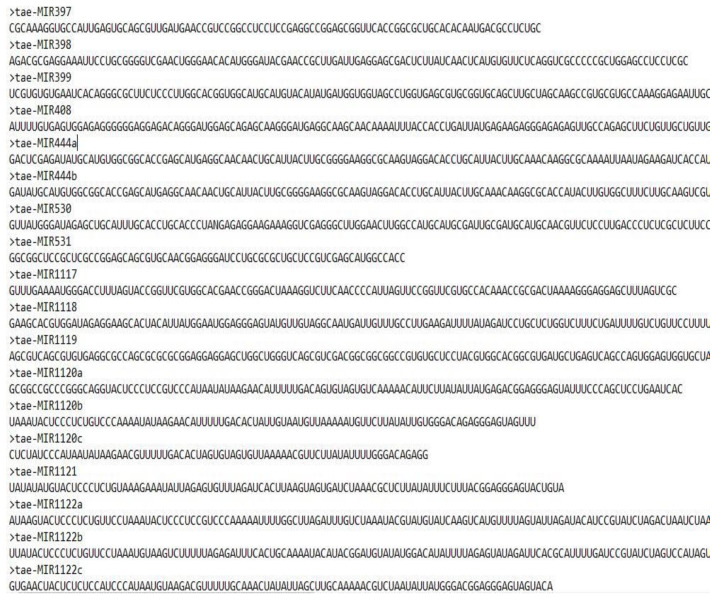
Sample of the dataset used in the experiments.

The experiment used the common wheat (Triticum aestivum) dataset, as shown in Figure 4. We downloaded 384 mature wheat miRNA sequences with high credibility from PNRD [46] and 19,011 wheat lncRNA sequences from GreeNC [47] because there is no public database of miRNA and lncRNA interaction pairs. As seen in Table 2, the identical sequences were eliminated, leaving 298 miRNAs and 18,468 lncRNAs.

### 7.2. Data Preprocessing

In this study, the miRNA–lncRNA interaction prediction tool used was psRNATarget. The sequences of target genes that can interact with miRNA were found by examining the degree of matching between miRNA and target sequences in plants. The filtered lncRNAs and miRNAs were then entered into the psRNATarget program for prediction, yielding a positive dataset of 18,468 miRNA–lncRNA interaction pairings. The construction of a negative dataset with substantial interference capabilities is required to validate the model’s performance further. A small percentage of miRNAs participate in interaction pairings due to their low and short sequence lengths; a small percentage of miRNAs participate in interaction pairings due to their low and short sequence lengths; consequently, the experiment mostly analyzed lncRNA sequences. To begin, whole lncRNAs were sorted into two groups: those that participated in this interaction and those that did not. Then, using the Needleman Wunsch algorithm [48], a similarity comparison between the two groups for lncRNA was performed, and samples of lncRNAs with similarities of more than 80% were eliminated [49].

After similarity elimination, lncRNAs that were not engaged in the lncRNA–miRNA interaction were randomly paired with all miRNAs to produce the negative sample datasets. A random sampling approach was applied to obtain the same numbers for negative samples as positive samples to guarantee the balance of negative and positive samples. The positive and negative datasets were jumbled randomly to create the 39,593 data points needed for the experiment. We employed the SMOTE method [50] to enhance the sample size by producing characteristic data that resemble the samples to address data insufficiency and small sample size issues. We randomly selected an eigenvalue from a positive sample, calculated the eigenvalue of the closest positive sample, and then created new positive samples between the two using positive samples as an example. We iterated the previous steps until the sample data were large enough. Because the dataset’s maximum sequence length exceeds 8000 nt, the training phase takes a long time. At the same time, there were just 315 sequences longer than 4000 nucleotides. As a result, we discarded sequences that were longer than 4000 nt. The findings show that after deleting data with sequence lengths of more than 4000 nt, CNN-RNN accuracy did not improve much, but the training time was considerably reduced. The original dataset is dataset 1, and dataset 2 is updated after deleting the data with sequence lengths of more than 4000 nt. Three experiments were carried out, which are presented in Table 2. Although the accuracy of CNN-RNN changed somewhat, the time of training for each batch was reduced by more than half.

### 7.3. k-mer Features of miRNA Sequence

*Triticum aestivum* features were extracted using a hybrid CNN-RNN model. The experiments used tenfold cross-validation to ensure the accuracy and dependability of the data. The experimental dataset was divided into ten groups: nine for training and one for verification. The medium values of 10 experiments are used as the final results after experimenting 10 times alternatively. The main extracted feature and secondary extracted feature of the sequence are the key features retrieved in this experiment. The most prevalent extracted feature is k-mer. Each k-mer contains nucleotides K that can be A, T, C, or G. The experiments extract sequence characteristics from 3-mer (64 dimensions), 2-mer (16 dimensions), and 1-mer (4 dimensions). To match the above k-mer, a sliding window with a length of k and a sliding step size of one is employed. The experiment also retrieved the sequence’s gap features, such as the initial gap feature (A*A, 64 dimensions) and the second gap feature (A**A, 256 dimensions).

Secondary structural features decided the primary functions of RNA molecules. According to studies, the more stable an RNA sequence’s structure leads to more free energy is produced during folding to build secondary structures; the more stable the secondary structure is the additional complimentary basis pairing it creates, with higher G and C values. This experiment extracted the sequences’ basis complimentary pairing rate (*E*_1_) and C and G values (*E*_2_), and normalized minimum free energies (*DM*). The ViennaRNA [51] toolbox was used to identify the point bracket form for the secondary sequence structures, as well as the least free energy created through the production of this secondary structure, which is characterized as follows:

(14) DM=MFE/L

(15) $$E_{1}=n\_pairs∕\left(L∕2\right)$$

(16) $$E_{2}=\left(n\_G+n\_C\right)∕L$$

where n_pairs is the maximum number of base pairs that may be paired at the sequences, *L* is the length of the sequence, n_C and n_G are the frequency occurrences for *C* and *G*, and *MFE* is the minimal free energy for the sequence.

There were 485 dimensions derived, covering both fundamental and secondary structural elements. The 485-dimensional feature vectors were created by fusing these features. Every feature vector was concatenated at vector sets for model testing and training. Table 3 shows the complete feature information.

The experiments also used tenfold cross-validation, with 90% of the data being used for training and 10% for testing. On the Triticum aestivum dataset, CNN-RNN is first compared to shallow machine learning approaches, including traditional machine learning algorithms such as random forest, k-nearest neighbor (k-NN), and support vector machine (SVM). Although deep learning harvests information automatically, the important features may be lost in the process, resulting in a generic and not optimum condition. As a result, deep learning approaches may not perform as well as shallow machine learning models.

**Figure 5:**
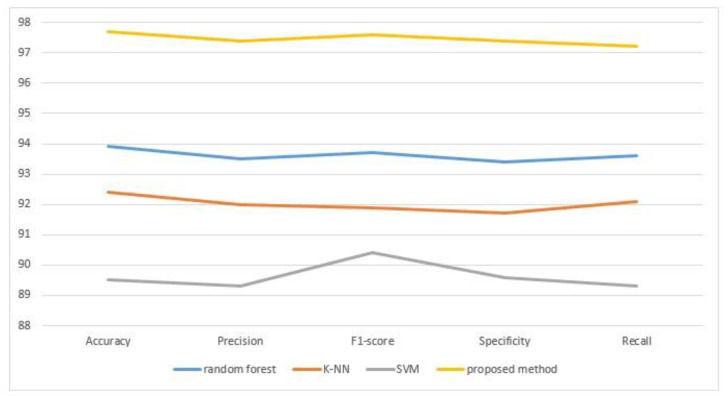
The performance metrics for shallow machine learning compared with our proposed method.

The proposed model was compared to shallow machine learning models and another deep learning model to verify its performance. Table 4 and Figure 5 demonstrate the experimental results of our suggested model and the shallow machine learning models. Table 4 shows that our suggested model achieves greater than 96% for all four assessment factors; clearly, this is higher than other models, demonstrating that our proposed model outperforms shallow machine learning approaches. Experimental data suggest that our proposed model outperforms shallow machine learning in the categorization of miRNA–lncRNA interactions.

**Figure 6:**
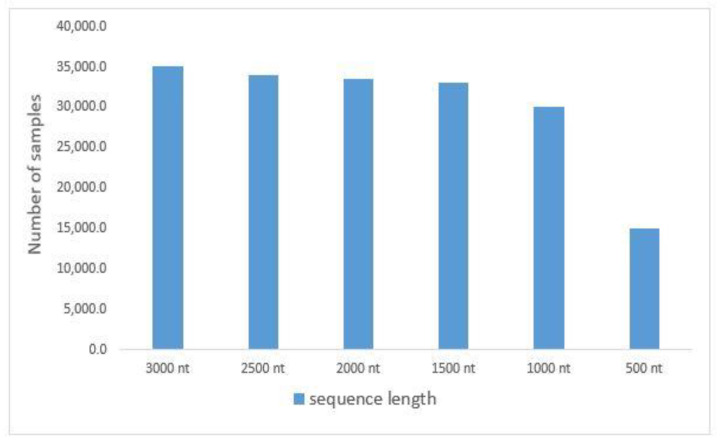
The distribution of our dataset.

The proposed model was compared to various deep learning models such as LSTM, IndRNN, CNN, and CNN+LSTM, and shallow machine learning methods. Each model was trained and tested using six sets of data and tenfold cross-validation; accuracy was utilized as the assessment criterion. The Triticum aestivum dataset is divided into six groups, with maximum sequence lengths of 3000 nt, 2500 nt, 2000 nt, 1500 nt, 1000 nt, and 500 nt for each group. Figure 6 depicts the data distribution. Table 5 shows the categorization findings.

**Figure 7:**
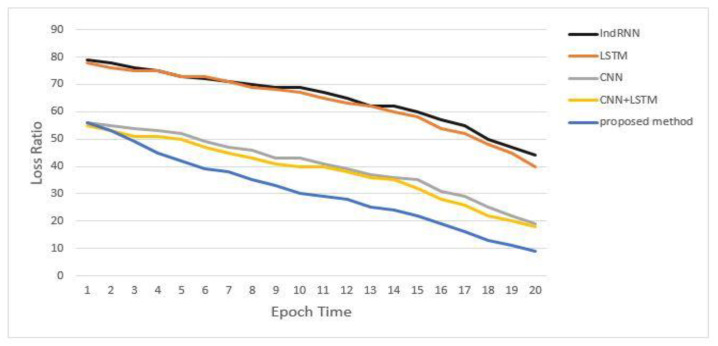
Loss ratio comparison of our proposed method with other different deep learning models.

Table 5 shows that the LSTM accuracy dramatically reduces as sequence length increases, whereas the CNN+LSTM accuracy marginally decreases. Only the accuracy of the proposed model and CNN remained unchanged, but our proposed method’s accuracy is substantially greater than CNN’s. The findings suggest that our proposed method outperforms previous deep learning models regarding miRNA–lncRNA interaction accuracy, particularly when the length of the sequences is rather large. We examined the loss convergences rates for the models when the length of the sequences is 3000 nt to test our model’s performance further. The loss convergence rate in 20 iterations is compared in Figure 7. In terms of both convergence rate and degree of convergence, our suggested strategy outperforms existing deep learning models.

In recent years, much work has been devoted to creating computer approaches for finding connections in diverse biological datasets. Many researchers have used shallow machine learning methods to construct a prediction model through feature selection in the prediction of the interaction between miRNA and lncRNA, but there are many problems such as fewer training data, large noise, and more human factors, resulting in low reliability of the prediction results. The comparative analysis of the proposed model with state-of-the-art models showed that the proposed model has better performance, with accuracy of 97.7%, greater than the models described in [5,10,17,19,21,22], as shown in Table 6. Additionally, we compared our model with the XGBoost model. We applied the same dataset in this model after comparison, and our model was slightly better than the XGBoost model, as shown in Table 7.

As shown in Table 7, the proposed model was compared with another advanced model, XGBoost model, to prove its effectiveness. We applied the same dataset used in this work to the XGBoost model for the comparison. The proposed model was slightly better than the XGBoost model in terms of accuracy, F1-score, recall, specificity, and precision. The results indicate that the proposed model is slightly better than the XGBoost method.

## 8. Conclusions

Based on the RNA sequence properties for plants, we propose a technique to predict the interaction between miRNA and lncRNA. The model efficiently solved the difficulties of gradient disappearance and explosion during the gradient propagation process and ensured classification accuracy. Furthermore, the model has a basic structure, is simple to use, and can be extended. Our suggested technique effectively classifies plant interactions between miRNA and lncRNA. The model has apparent benefits over shallow machine learning and other deep learning models, and it may be extended to other plants with acceptable results. At the same time, the model has a high level of performance and generalization, making it helpful in classifying plant miRNA–lncRNA interactions. This study has laid the groundwork for further investigation into the interaction mechanism of miRNA and lncRNA in plants. Model classification accuracy could be further enhanced in the future by modifying the level of model structures and increasing datasets.

In future work, we will apply our proposed model to other datasets in various fields, especially agriculture. Furthermore, it would be interesting to study the influence of combining additional deep learning models or using different optimization models.

## Figures and Tables

**Table 1:** Suggested model parameter settings.

Hyperparameters	Value
Optimization algorithm	GTO
Initial learning rate	0.1
Dropout layer	0.5
Epochs	50
Batch size	128

**Table 2:** Wheat (Triticum aestivum) dataset information.

Datasets	Original Data	Filtered Data	Databases
lncRAN	19,011	18,468	GreeNC [36]
miRNA	384	298	PNRD [35]

**Table 3:** Information on structural features.

Feature Category	Feature Name	Number
Primary structural features	1-mer	4
	2-mer	16
	3-mer	64
	First gap features	64
	Second gap features	256
Secondary structural features	DM	1
	$E_{1}$	1
	$E_{2}$	1

**Table 4:** The performance metrics of shallow machine learning models compared with our proposed method.

Performance Measures	Accuracy	Precision	F1-Score	Specificity	Recall
random forest	93.9	93.5	93.7	93.4	93.6
K-NN	92.4	92	91.9	91.7	92.1
SVM	89.5	89.3	90.4	89.6	89.3
proposed method	97.7	97.4	97.6	97.39	97.21

**Table 5:** Comparison analysis of the proposed models with different lengths of sequences and different deep learning models.

Models	Length of Sequences					
	3000 nt	2500 nt	2000 nt	1500 nt	1000 nt	500 nt
IndRNN	76.49	76.37	76.53	76.58	76.97	76.76
LSTM	69.98	71.56	73.86	76.34	79.64	83.74
CNN	95.29	94.32	95.51	94.91	94.67	95.25
CNN+LSTM	95.46	95.76	95.25	95.71	95.78	96.41
Proposed method	96.45	96.60	97.21	97.85	97.33	97.7

**Table 6:** Comparison analysis of the proposed model.

Year	Existing Work	Accuracy %
2022	X Du et al. [5]	92
2022	Chen, Lin et al. [10]	91.9
2019	Chen, Kunqi et al. [22]	80
2023	Proposed model	97.7

**Table 7:** Comparison of the suggested model with the XGBoost model.

Performance Measures	Accuracy	Precision	F1-Score	Specificity	Recall
XGBoost	96.5	94.3	96.4	92.6	93.3
Proposed method	97.7	97.4	97.6	97.39	97.21

## Data Availability

The data presented in this study are available on request from the corresponding author.

## Abbreviation

SVM	Support Vector Machines
BLSTM	Bidirectional Long Short-Term Memory
KNN	K-Nearest Neighbors
CNN	Convolutional Neural Network
RNN	Recurrent Neural Network
IndRNN	Independently Recurrent Neural Network
SMOTE	Synthetic Minority Oversampling Technique
LSTM	Long Short-Term Memory Networks
